# The Effect of Increasing the Body’s Core Temperature and Improving Blood Flow by Using Far-Infrared Rays Emitted from Functional Loess Bio-Balls

**DOI:** 10.3390/biomedicines12122922

**Published:** 2024-12-23

**Authors:** Yong-Il Shin, Min-Seok Kim, Yeong-Ae Yang, Gye-Rok Jeon, Jae-Ho Kim, Yeon-Jin Choi, Woo-Cheol Choi, Jae-Hyung Kim

**Affiliations:** 1 Department of Rehabilitation Medicine, School of Medicine, Pusan National University, Yangsan 50612, Republic of Korea; rmshin@pusan.ac.kr; 2 Department of Rehabilitation Medicine, Yangsan Hospital, Pusan National University, Yangsan 50612, Republic of Korea; 3 Monash Health, Melbourne, VIC 3800, Australia; minseok.kim@monashhealth.org; 4 Department of Occupational Therapy, Inje University, Gimhae 50834, Republic of Korea; otyya62@inje.ac.kr; 5 R&D Center, eXsolit, Yangsan 50611, Republic of Korea; grjeon@pusan.ac.kr (G.-R.J.); jhkim@pusan.ac.kr (J.-H.K.); 6 R&D Center, Hanwool Bio, Yangsan 50516, Republic of Korea; hbi1004@naver.com (Y.-J.C.); lih1769@naver.com (W.-C.C.)

**Keywords:** loess bio-ball, far-infrared rays (FIRs), body’s core temperature, thermoregulation, blood circulation

## Abstract

Background: Low-energy far-infrared rays (FIRs) are widely used in the treatment of wounds, lymphedema, and various vascular diseases, and various types of products that emit infrared rays are being used at home for patients with blood flow-related diseases without experimental evidence. Methods: Blood flow and epidermal temperature were measured while applying conductive heat and FIRs via an electric mat (non-intervention) or a loess bio-ball mat (intervention). Results: In the control group (*n* = 30), there was a minimal change in blood flow and epidermal temperature in the right and left middle fingers (LMF, RMF) as the mat temperature gradually increased. In the experimental group (*n* = 30), when the mat temperature increased from 25 °C to 50 °C, the blood flow increased by 39.80% in the LMF and by 41.83% in the RMF. In addition, the epidermal temperature increased by 8.78% in the LMF and by 8.44% in the RMF. Conclusions: The FIRs emitted from loess bio-balls can be applied to alleviate symptoms not only in patients with blood flow problems in medical settings but also in people who complain of discomfort due to blood flow disorders or cold hands and feet during their daily life and sleep.

## 1. Introduction

In far-infrared therapy, far-infrared rays (FIRs) penetrate the epidermis to increase the body’s core temperature, improve blood flow, and activate various metabolic functions [1]. FIRs can reach 4–5 cm into the epidermis and are absorbed by tissues, increasing the vibrational motion of water molecules and generating heat [2]. FIRs with wavelengths of 4–16 μm non-specifically reduce the size of water clusters in vivo and enhance fluid motility [3]. As their frequency is in the same range as the natural frequency of water molecules, their effect can be further amplified in tissues through the resonance effect [4]. FIRs have many therapeutic effects and can be used for disease and symptom management [5,6]. They can reduce muscle damage and improve running recovery [7]. In patients with peripheral vascular disease, FIRs significantly improve surface temperature, blood flow, and plantar pressure [8]. Similarly, FIRs increased epidermal temperature and improved sensitivity to pain, touch, and pressure in patients with diabetic foot disease. In lymphedema, FIRs and compression bandaging reduce edema and tissue fibrosis and improve skin elasticity [9]. FIRs have also been shown to improve the microcirculation and optimize cellular metabolic activity [10]. Low-energy FIRs have been widely applied in the management of vascular-related diseases [10,11]. Therefore, a systematic analysis of two comparative experiments (with conductive heat and FIRs) is needed, utilizing the energy conversion of FIRs and the mechanism of action of body temperature regulation.

Loess has long been used for therapeutic purposes owing to its health-promoting effects. Functional loess bio-balls manufactured using the low-temperature method retain useful enzymes and minerals and emit FIRs in the 5–20 μm wavelength range. The frequency of the FIRs emitted from loess is similar to that of the energy absorbed by cellular water molecules [2]. FIR energy is converted into vibrational energy in water molecules, which can increase the body’s core temperature and contribute to blood circulation. The remaining energy can activate cells through non-thermal processes [4]. In this study, the effects of FIRs on blood flow and skin temperature enhancement were quantitatively compared and analyzed using a conventional electric mat (non-FIR treatment) and a loess bio-ball mat (FIR treatment).

## 2. Materials and Methods

### 2.1. Mechanisms of Thermoregulation in the Body

**Figure 1:**
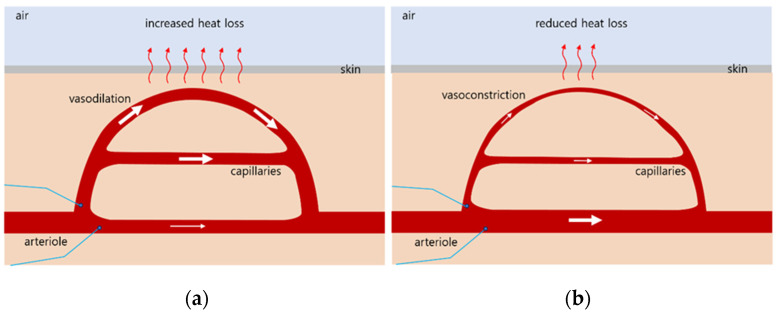
Hypothalamic thermoregulatory mechanism. (**a**) When the body’s core temperature is above 37 °C; (**b**) when the body’s core temperature is below 37 °C. The white arrows indicate the direction and amount of blood flow, and the red arrows indicate the direction and amount of heat loss.

The body temperature is regulated by sensing the blood temperature around receptors in the hypothalamus [12,13]. Through the thermoregulatory center in the hypothalamus, a temperature of 37 °C (98.6 F) is maintained, which is optimal for enzyme function [14,15]. The skin also contains thermo-receptors that send feedback to the thermoregulatory center. When the body’s core temperature rises above 37 °C, the receptors signal the hypothalamus, which transmits nerve impulses to the arterioles of the skin, causing vasodilation and increased blood flow (Figure 1a) [16]. When the temperature falls below 37 °C, the receptors signal the hypothalamus, which then transmits nerve impulses to the arterioles in the skin, causing vasoconstriction and reduced capillary blood flow (Figure 1b).

### 2.2. Study Participants

The call for the recruitment of subjects for clinical research on blood circulation was published as an article in the local newspaper “Yangsan News Park”, dated 7 November 2023. As for the eligibility criteria for participation, we included subjects who were 30 years or older and regularly experienced discomfort due to cold hands and feet or blood flow disorders. None of the participants were on medications that could affect the autonomic nervous system (ANS) function. Full consent was obtained from all participants after the study was explained. The demographic and clinical characteristics of the 60 participants who ultimately participated in the experiment and analysis are presented in Table 1. The participants were recruited among people who complained of disability or discomfort due to poor blood flow. For the experimental group (14 females, 16 males), the age was 60.17 (±10.35) years, and the BMI was 21.40 (±3.01) kg/m^2^. For the control group (16 females, 14 males), the age was 57.44 (±8.53) years, and the BMI was 24.77 (±3.26) kg/m^2^.

### 2.3. Trial Design and Setting

Blood flow and epidermal temperature were measured while the participants were comfortably lying on an electric mat that did not emit FIRs or on a loess bio-ball mat that emitted FIRs. The temperature range of the cushion placed on the mat on which the participants lay was 30 °C to 37 °C. The wavelength of the laser diode used for measuring the blood flow was 780 nm, and the output was approximately 2 mW. Therefore, it was unlikely to have any harmful effects on the participants in this experiment. Participants who moved their bodies frequently or complained of discomfort during the measurement were excluded, and the experiment was continued with additional participants.

**Figure 2:**
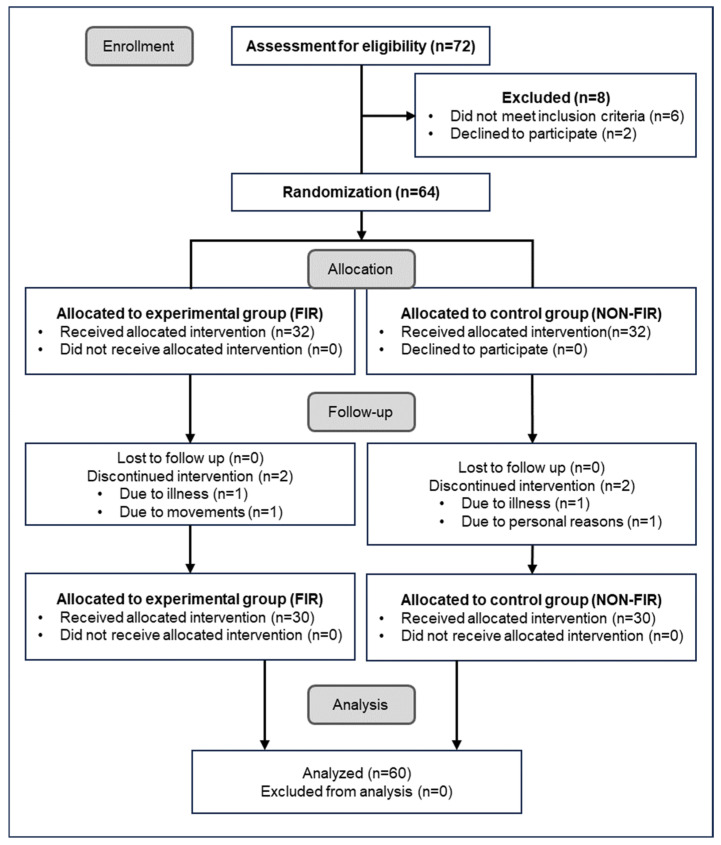
CONSORT diagram of enrollment, participation, and experimental data analysis.

The experimental protocol was approved by the Inje University Bioethics Committee (registration number: INJE 2023-05-035-005, dated 20 September 2023), under the clinical trial registration entitled “Improvement of blood circulation by far-infrared rays emitted from loess bio-balls and health promotion effects in related diseases”. This study was conducted at Hanwool Bio Lab from 7 November 2023 to 15 January 2024, based on the Consolidated Standard of Reporting Trials (CONSORT), as shown in Figure 2 [17].

The exclusion or discontinuation criteria for the intervention were as follows: pregnant women or applicants with cancer, diabetes, cardiovascular, or neurological diseases. The participants who showed symptoms of mental weakness, anxiety, or mental illness (including dementia) or who withdrew from participation due to illness or personal reasons were also excluded from the intervention. Of the 72 participants, 6 subjects were excluded because they did not meet the inclusion criteria, and 2 subjects withdrew from participating in the study for personal reasons, resulting in a total of 8 sub-jects being excluded. After hearing an explanation of each experiment, the 64 participants expressed their willingness to participate in each experiment, taking into account their personal circumstances. The participants selected a sealed envelope (A or B) received from the research manager and were randomly assigned to the experimental (A) and control (B) groups. From the time of intervention allocation, the measurers, data analysts, and outcome evaluators were blinded to the subjects and the trial details. During the follow-up experiment, 4 additional subjects were excluded due to illness (*n* = 1) and frequent movements (*n* = 1) in the experimental group A and illness (*n* = 1) and personal reason (*n* = 1) in the control group B. The total number of subjects in group A (*n* = 30) and group B (*n* = 30) participated in each experiment, and the experimental results were analyzed.

### 2.4. Interventions

**Figure 3:**
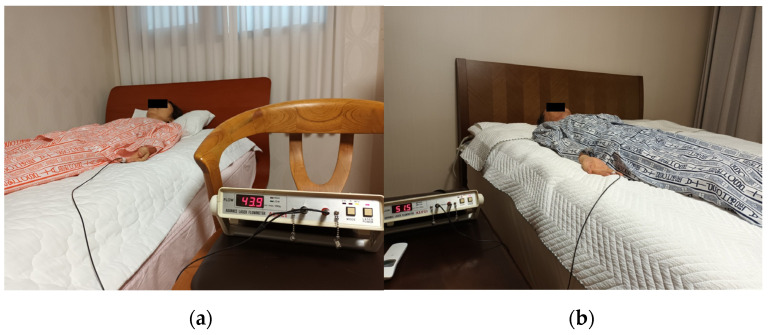
Participants’ lying postures and electrode attachment location for blood flow measurements on (**a**) electrical mat and (**b**) loess bio-ball mat.

A conductive electric mat (SW-301, SHS, Samhwa Electric Blanket, Pusan, Republic of Korea) was used in the control experiment. The temperature was set at 3 °C intervals from 25 °C to 49 °C. For the experimental group, a Jangsoo bio-ball bed (7111, Jangsoo Industry Co. Ltd., Seoul, Republic of Korea) with a radiant intensity of 3.74 × 10^2^ W/m^2^ around 9.5–9.8 μm was used as an FIR source at 2.5 °C intervals from 25 °C to 50 °C. Blood flow was measured using a Doppler laser flowmeter (ALF21; Advanced Co., Ltd., Tokyo, Japan). The red diode laser (780 nm, 2 mW) applied to the reflective electrode could penetrate the skin to a depth of approximately 0.5–1 mm, and the electrode was attached to the middle finger (LMF, RMF) using a bandage. The epidermal temperature was measured using an infrared thermometer (MM-GP100, Harbin Xiande Technology Development Co., Ltd., Shenzhen, China). Data analysts (evaluators) performed the task without knowing the participants’ health status or the type and condition of the mats. Figure 3 shows the subjects participating in the blood flow measurement while lying on (a) an electric mat and (b) a loess bio-ball mat with electrodes attached to the middle fingers.

### 2.5. Experimental Measurements

#### 2.5.1. Temperature Changes in the Cushion Placed on the Electric Mat and Loess Bio-Ball Mat

In the control group, 30 subjects were placed in a supine position on an electric mat with a cushion with thickness of 1 cm. The temperature of the electric mat was set at 3 °C intervals from 25 °C to 49 °C. The set temperature delivered to the electric mat might differ from the temperature received by the participant. When an electric mat is used, heat can be transferred to the body surface via thermal conduction. For the experimental group, the heat (expressed as the set temperature) applied to the loess bio-ball mat and the temperature actually received by the participants might also be different. Therefore, the relationship between the set temperature applied to the bio-ball mat and the temperature of the cushion was investigated by increasing the setting temperature at 2.5 °C intervals from 25 °C to 50 °C. The temperature of the cushion (approximately 1 cm thick) placed on the loess bio-ball mat was measured while applying heat to the mat wire.

#### 2.5.2. Blood Flow

In the control group, blood flow in the LMF and RMF was measured using a laser Doppler flowmeter, while heat (set temperature) was applied to the electric mat from 25 °C to 50 °C at 3 °C intervals. For the experimental group, blood flow was investigated using a laser doppler flowmeter in the LMF and RMF while applying heat (set temperature) to the loess bio-ball mat at 2.5 °C intervals from 25 °C to 50 °C. The wavelength (9.5–9.8 μm) of the FIRs emitted from the loess bio-ball is close to the absorption wavelength of water molecules vibrating in the body [18,19]. Therefore, when the temperature of the loess bio-ball is increased, the vibrating water molecules absorb more FIR energy, which results in active vibrations.

#### 2.5.3. Epidermal Temperature

As the body’s core temperature increases, the blood flow increases because of the body’s thermoregulatory mechanism. In addition, as the blood flow increases, the epidermal temperature increases at the skin surface, where many capillaries are distributed. In this experiment, the epidermal temperature in the LMF and RMF was measured using a non-contact infrared thermometer while increasing the set temperature of the electric and loess ball mats.

### 2.6. Statistical Analyses

For the control group (*n* = 30) and the experimental group (*n* = 30), the correlation coefficient (*r*) and *p*-value were calculated using IBM-SPSS Statistics (version 29.0.2.0; IBM Corp., SPSS Inc., Armonk, NY, USA) to calculate blood flow and epidermal temperature measured in the LMF and RMF at each temperature. A *p*-value of <0.001 was considered statistically significant. Data processing, graphing, and logistic fitting were performed using Microsoft Excel 2016 (Microsoft Corp., Redmond, WA, USA).

## 3. Results

### 3.1. Temperature Change on the Cushion When Heat Was Applied to the Electric Mat and Loess Bio-Ball Mat

**Figure 4:**
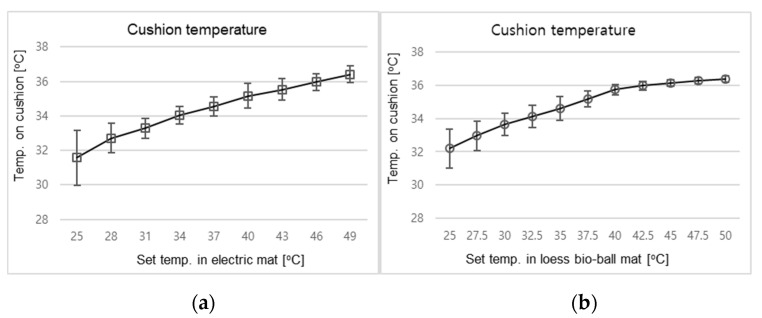
Temperature on the cushion as heat was applied to (**a**) the electric mat and (**b**) the loess bio-ball mat. In the figure, temp. is an abbreviation for temperature. The error bars represent the standard deviation of the temperatures measured on the cushions placed on the electric mat and the loess bio-ball mat.

Figure 4a shows the temperature measured 20 times on the cushion placed on the electric mat as heat (indicated by the set temperature) was applied to the mat. When the set temperature applied to the mat was increased from 25 to 49 °C, the temperature measured on the cushion increased from 31.58 °C to 36.41 °C. Figure 4b shows the temperature measured 20 times on the cushion placed on the loess ball mat as heat (indicated by the set temperature) was applied to the mat. When the set temperature was increased from 25 °C to 50 °C, the temperature on the cushion increased from 32.19 °C to 36.37 °C. As shown in Figure 4, the temperature of the cushions placed on the electric mat and loess ball mat showed a similar increase depending on the applied heat. In the experiments with the control and experimental groups, the participants were expected to have blood flow measurements performed under similar temperature conditions without temperature-related risks.

### 3.2. Changes in Blood Flow and Epidermal Temperature Measured in the LMF and RMF When Using the Electric Mat and the Loess Bio-Ball Mat

Table 2 shows blood flow and epidermal temperature measured in the LMF and RMF when using an electric mat. When the set temperature applied to the electric mat was increased from 25 °C to 49 °C, there was a minimal change in blood flow (0.35 mL/min, 0.91%) in the LMF. The epidermal temperature measured in the LMF showed a slight change of 0.46 °C (1.35%). In addition, there was also a minimal change in blood flow (0.39 mL/min, 1.02%) in the RMF. The epidermal temperature measured in the RMF showed a slight increase of 0.46 °C (1.35%).

Table 3 shows blood flow and epidermal temperature measured in the LMF and RMF when using a loess bio-ball mat. When the set temperature applied to the loess bio-ball mat was increased from 25 °C to 50 °C, the blood flow in the LMF significantly increased by 15.18 mL/min (39.80%), from 38.14 ± 7.30 mL/min to 53.32 ± 7.51 mL/min. The epidermal temperature measured in the LMF gradually increased by 2.96 °C (8.78%), from 33.72 ± 1.02 °C to 36.68 ± 0.25 °C. In addition, the blood flow in the RMF increased by 16.02 mL/min (41.83%), from 38.30 ± 5.45 mL/min to 54.32 ± 7.27 mL/min, depending on the set temperature. The epidermal temperature measured in the RMF gradually increased by 2.86 °C (8.44%), from 33.87 ± 1.40 °C to 36.73 ± 0.21 °C. The Pearson correlation coefficient was 0.785–0.998 for the LMF and 0.497–0.996 for the RMF, showing a significant difference. This suggests that when the temperature of the loess bio-ball mat increased, there was a significant difference in the blood flow of the experimental subjects.

In particular, the *p* value for the blood flow between 25 °C and 50 °C in the RMF was high, being 0.025.

Accordingly, the standard deviation was relatively large, being ±6.64~±7.51 in the LMF and ±5.45~±7.87 in the RMF.

### 3.3. Blood Flow and Epidermal Temperature According to the Set Temperature in the LMF When Using the Electric Mat and the Loess Bio-Ball Mat

**Figure 5:**
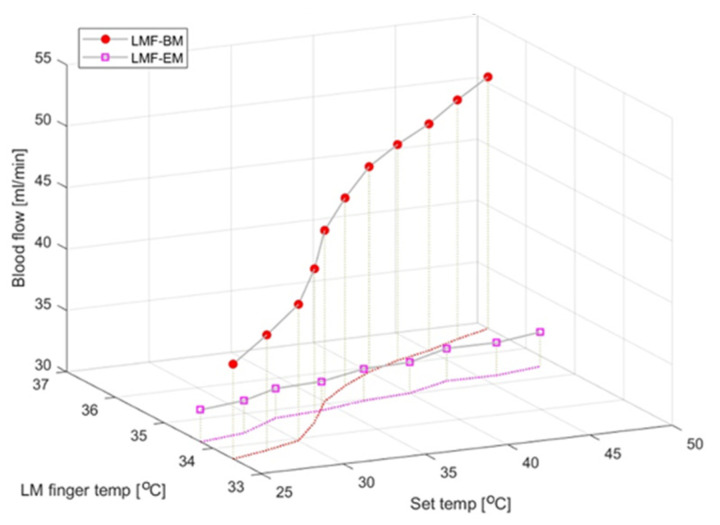
Changes in blood flow and epidermal temperature in LMF according to set temperature for 60 participants on electric mat and loess bio-ball mat. Since the temperature differences at the skin surface depending on the applied heat and FIR (expressed as the set temperature) are small, the temperatures are represented as shapeless pink and red dotted lines to clearly show these differences. temp is an abbreviation for temperature.

Figure 5 shows the blood flow and epidermal temperature in the LMF according to the set temperature when using the electric and loess bio-ball mats. To simplify the figure, only the average values of blood flow and epidermal temperature according to the set temperature are shown, excluding the standard deviation. There was a minimal change in blood flow on the electric mat (pink squares), but the blood flow significantly increased on the loess bio-ball mat (red circles). This is attributed to an increase (39.80%) in the blood flow owing to the FIRs emitted from the loess bio-balls. In addition, the pink dotted line represents the change in epidermal temperature when using the electric mat, and the red solid line represents the change in epidermal temperature when using the loess ball mat. While the epidermal temperature increased by 0.46 °C (1.35%) on the electric mat, it increased by 2.96 °C (8.78%) on the loess bio-ball mat. The increase indicated by the red solid line is believed to be caused by the FIRs emitted from the loess bio-balls.

### 3.4. Blood Flow and Epidermal Temperature According to the Set Temperature in the RMF When Using the Electric Mat and the Loess Bio-Ball Mat

**Figure 6:**
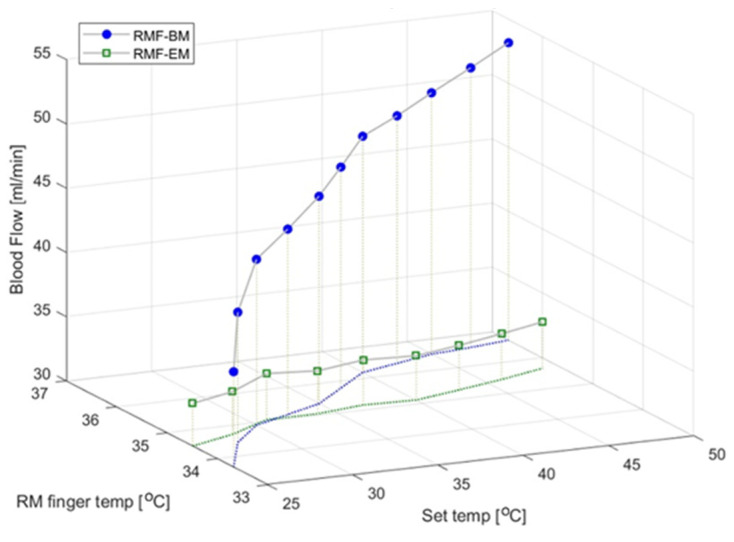
Changes in blood flow and epidermal temperature in the RMF depending on the set temperature of the electric mat and loess bio-ball mat.Since the temperature differences at the skin surface depending on the applied heat and FIR (expressed as the set temperature) are small, the temperatures are represented as shapeless green and blue dotted lines to clearly show these differences. temp is an abbreviation for temperature.

Figure 6 shows the blood flow and epidermal temperature in the RMF at the set temperature when using the electric and loess bio-ball mats. For simplicity, only the average values of blood flow and epidermal temperature according to the set temperature are shown, excluding the standard deviations. There was a minimal change in the blood flow on the electric mat (green squares), but the blood flow significantly increased on the loess bio-ball mat (blue circles). This was attributed to an increase (41.83%) in the blood flow owing to the FIRs emitted from the loess bio-balls. The green dotted line represents the change in epidermal temperature in the RMF when using the electric mat, and the blue solid line represents the change in epidermal temperature in the RMF when using the loess ball mat. While the epidermal temperature increased by 0.46 °C (1.35%) on the electric mat, it increased by 2.86 °C (8.44%) on the loess bio-ball mat. The increase indicated by the blue solid line is attributed to the FIRs emitted from the loess bio-balls.

### 3.5. Scatter Plot Showing the Slope and Offset for Blood Flow in the LMF and RMF for 30 Control and 30 Experimental Subjects

**Figure 7:**
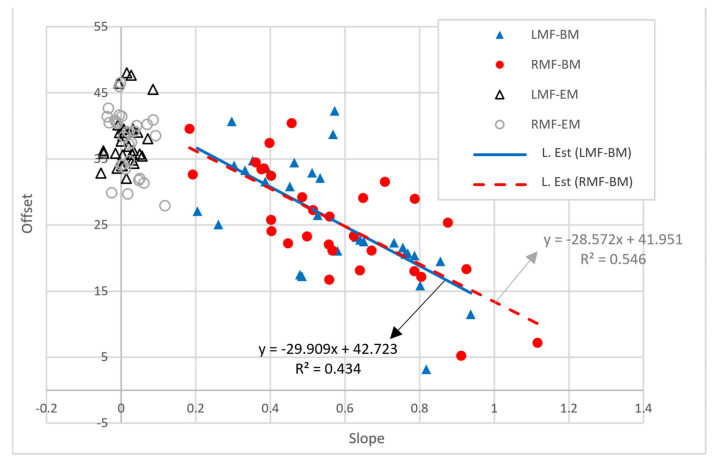
Scatter plot showing the slope and offset of blood flow in LMF and RMF. BM stands for electric mat, and LM stands for loess bio-ball mat. L. Est represents the estimated line.

Figure 7 shows a scatter plot (slope vs. offset) for the LMF and RMF for the 60 participants of the study. Here, the slope represents the change in blood flow according to the individual temperature setting, and the offset represents the intercept for blood flow on the *y*-axis. In the control experiments (LMF-EM and RMF-EM), the black triangles and gray circles are concentrated on the *y*-axis, confirming a minimal increase in blood flow. However, for the experimental group (LMF-BM and RMF-BM), the blue triangles and red circles are widely spread, and the offset decreases as the slope increases.

The patterns for the LMF-BM (blue triangles) and RMF-BM (red circles) are slightly scattered around the estimated lines (blue solid, red dotted). The blue solid line (L. Est: LMF-BM) represents the distribution (blue triangles) of the offsets for the slopes for the blood flow in the LMF for the 30 participants. The blue solid line (LMF-BM) is an estimated line (*y* = −29.909*x* + 42.723; *R*^2^ = 0.434), indicating an offset for the slope for the LMF. The red dotted line (L. Est: RMF-BM) is an estimated line showing the distribution (red circles) of the offsets versus the slope for the blood flow in the RMF for the 30 participants. The red dotted line (RMF-BM) is an estimated line (*y* = −28.572*x* + 41.951; *R*^2^ = 0.546) representing the offset for the slope for the RMF.

In the scatter plot in Figure 7, the black triangles and gray circles are constrained to a slope of approximately = 0. This implies that the blood flow did not increase depending on the set temperature applied to the electric mat. However, the blue triangles and red circles are widely distributed around the solid and dotted lines, respectively. This means that when the set temperature of the loess bio-ball mat was increased, the blood flow measured in the participant’s LMF and RMF increased proportionally to the slope. In addition, the negative estimated lines in the scatter plot indicate that when the blood flow was high, the increase in blood flow was small, whereas when the blood flow was low, the increase in blood flow was large.

## 4. Discussion

Loess has been used in various forms for therapeutic purposes [19]. According to the IR absorption spectrum, unlike high heat-treated loess, loess bio-balls manufactured using the low-temperature wet-drying method can maintain the useful components of raw loess [20]. They emit a large amount of FIRs due to the vibrational motion (stretching) of Si-O around 9.5–9.8 μm. The frequency of the FIRs emitted from loess bio-balls matches the absorption frequency of water molecules; therefore, water molecules in the body can selectively absorb FIR energy, thereby increasing the body’s core temperature [4]. When the body’s core temperature rises above 37 °C, the hypothalamic thermoregulatory mechanism leads to vasodilation and increases the blood flow to the capillaries [21]. The unique physical properties of the FIRs emitted from loess bio-balls can be exploited for therapeutic purposes.

Recent studies have indirectly measured the blood flow by measuring the skin temperature after irradiating FIRs to specific body’s parts, such as the feet and hands. These studies have mainly detected changes in biomarkers in the body before and after FIR application [4,11,22]. Far infrared ray (FIR) therapy offers an effective and oncologically safe treatment for breast cancer-related lymphedema [23]. FIR therapy can reduce fluid volume and limb circumference. Clinically, FIRs did not promote breast cancer recurrence, had no side effects, and did not promote cell proliferation; they also did not shorten the cell cycle of breast cancer cells in vitro. In a trial applying far-infrared therapy to the foot circulation of diabetic hemodialysis patients, significant positive effects on the temperature, pulse, and blood flow of the arteries in the dorsum of the foot were observed by FIR therapy [24].

In this study, changes in blood flow and epidermal temperature according to the set temperature were simultaneously measured while irradiating the entire body with FIRs emitted from loess bio-balls. Our findings showed that the effect of FIRs on blood flow was effectively reflected in real time rather than before or after FIR exposure [20]. The changes in blood flow and epidermal temperature were analyzed considering the energy conversion between FIRs and water molecules and the thermoregulatory mechanism of the hypothalamus. The FIRs emitted from loess bio-balls can activate cells and tissues through the vibrational resonance of water molecules, thereby activating the lymphatic circulation and helping discharge waste and toxins [25,26]. The relationship between FIR exposure and lymph drainage can be confirmed by examining bioimpedance parameters [27], which can determine phase angle (PA), extracellular water (ECW), intracellular water (ICW), total body water (TBW), and body cell mas (BCM) status before and after FIR irradiation [28,29,30]. While the blood flow responds to FIRs in real time, a long-term exposure to FIRs has been shown to have a slow (average speed of 0.9 mm/s) lymphatic circulation effect that can reduce inflammatory fluids in the body. Even when heat (up to 50 °C) is applied to loess bio-ball, the actual temperature on the skin is low, at around 36 °C, because the subject is on a cushion that is about 1–1.5 cm thick. Therefore, it is expected that there will be almost no side effects from far-infrared rays from loess bio-balls. In the experimental group exposed to FIRs emitted from a loess bio-ball mat at 30 °C for 7 h during sleep, bioimpedance values such as PA (+3.80%), ECW (−3.38%), ECW/ICW (−4.84%), ECW/TBW (−2.63%), and ECW/BCM (−2.33%) related to inflammatory fluid were significantly changed [31]. The application of FIRs emitted from loess bio-balls can improve the lymphatic circulation and reduce swelling and inflammation [32], which is expected to open new areas of exploration in the field of alternative and complementary medicine.

This study was conducted primarily on participants who complained of disability or discomfort due to impaired blood flow. If experiments are conducted in a more controlled environment targeting specific patient groups in specialized medical institutions, the results of this study could be applied to various medical environments in the future.

## 5. Conclusions

The FIRs emitted from loess bio-balls activated water molecules and increased the core temperature of the body. This significantly improved the blood flow through the thermoregulatory mechanism of the hypothalamus. In the control group (non-FIR), there was a minimal change in blood flow and epidermal temperature in the LMF and RMF as the mat temperature gradually increased. However, when the set temperature of the loess bio-ball mat was increased from 25 °C to 50 °C, the blood flow increased by 39.80%, from 38.14 ± 7.30 mL/min to 53.32 ± 7.51 mL/min, in the LMF and by 41.83%, from 38.30 ± 5.45 mL/min to 54.32 ± 7.27 mL/min, in the RMF. Additionally, the blood circulation increased the temperature of the epidermis, where many capillaries are distributed. The epidermal temperature increased by 8.78%, from 33.72 ± 1.02 °C to 36.68 ± 0.25 °C, in the LMF and by 8.44%, from 33.87 ± 1.40 °C to 36.73 ± 0.21 °C, in the RMF. The FIRs emitted from the loess bio-balls stimulated the water molecules in the body, increasing the body’s core temperature. Therefore, they effectively increased blood flow and epidermal temperature by improving the blood circulation through the body’s thermoregulatory mechanism. Therefore, the FIRs emitted from loess bio-balls can be utilized as a complementary and alternative therapy for diseases related to poor blood circulation.

## Figures and Tables

**Table 1:** Demographic characteristics and physical conditions of the recruited subjects (*n* = 60).

Variables		Experimental Group (*n* = 30)	Control Group (*n* = 30)
Gender	Female	14 (46.67%)	16 (53.33%)
	Male	16 (53.33%)	14 (46.67%)
Age		60.17 (±10.35)	57.44 (±8.53)
BMI [kg/m^2^]		21.40 (±3.01)	24.77 (±3.26)
Blood flow		Discomfort or disorder	Discomfort or disorder

BMI is an abbreviation for body mass index.

**Table 2:** Blood flow and epidermal temperature in the middle fingers at the set temperatures when using an electric mat.

Temperature [°C]		25	28	31	34	37	40	43	46	49
LMF	BF [mL/min]	38.27 ± 4.58	38.34 ± 4.46	38.53 ± 4.48	38.57 ± 4.85	38.57 ± 4.65	38.62 ± 4.97	38.62 ± 4.87	38.59 ± 4.87	38.62 ± 4.90
	Temperature. [℃]	34.07 ± 1.00	34.12 ± 1.02	34.28 ± 1.02	34.37 ± 1.02	34.47 ± 1.07	34.54 ± 1.12	34.54 ± 1.10	34.53 ± 1.16	34.53 ± 1.07
RMF	BF [mL/min]	38.25 ± 4.56	38.22 ± 4.60	38.57 ± 4.28	38.59 ± 4.47	38.62 ± 4.66	38.67 ± 4.49	38.63 ± 4.42	38.70 ± 4.29	38.64 ± 4.40
	Temperature. [℃]	34.17 ± 0.92	34.39 ± 0.91	34.47 ± 0.92	34.47 ± 0.93	34.49 ± 0.92	34.48 ± 0.91	34.52 ± 0.94	34.58 ± 0.94	34.63 ± 0.96

Correlation coefficient (*r*) for blood flow (BF) in LMF and RMF was 0.980–0.995 and 0.969–0.991, respectively. *p*-values < 0.001. *r* for epidermal temperature in LMF and RMF was 0.798–0.978 and 0.879–0.938, respectively. *p*-values < 0.001.

**Table 3:** Blood flow (BF) and epidermal temperature in the middle fingers at the set temperatures when using a loess bio-ball mat.

Temperature [°C]		25	27.5	30	32.5	35	37.5	40	42.5	45	47.5	50
LMF	BF [mL/min]	38.14 ±7.30	41.08 ±6.77	43.11 ±6.64	45.03 ±6.79	46.49 ±7.01	48.07 ±7.27	48.96 ±7.22	50.01 ±7.33	50.78 ±7.38	51.82 ±7.43	53.32 ±7.51
	Temperature. [℃]	33.72 ±1.02	34.10 ±0.97	34.43 ±0.89	34.69 ±0.86	35.00 ±0.76	35.34 ±0.67	35.73 ±0.51	36.00 ±0.43	36.27 ±0.34	36.50 ±0.25	36.68 ±0.25
RMF	BF [mL/min]	38.30 ±5.45	42.12 ±6.09	44.68 ±7.00	46.51 ±7.31	48.26 ±7.87	49.72 ±7.65	50.70 ±7.54	51.48 ±7.54	52.40 ±7.15	53.52 ±7.25	54.32 ±7.27
	Temperature. [℃]	33.87 ±1.40	34.61 ±1.24	35.01 ±1.13	35.33 ±1.02	35.63 ±0.93	35.92 ±0.59	36.20 ±0.43	36.37 ±0.31	36.53 ±0.25	36.63 ±0.24	36.73 ±0.21

*r* for BF in the LMF and RMF was 0.785–0.998 and 0.497–0.996, respectively. *p*-values < 0.001, except for *p*-value = 0.025 between 25 °C and 50 °C in the RMF. *r* for epidermal temperature in the LMF and RMF was 0.391–0.979 and 0.593–0.961, respectively. *p*-values < 0.001, except for *p*-value = 0.033 between 25 °C and 40 °C in the LMF.

## Data Availability

The data contain sensitive personal and physical information on the study participants and are available from the corresponding author upon reasonable request.
